# The Impact of Age and Cognitive Reserve on Resting-State Brain Connectivity

**DOI:** 10.3389/fnagi.2017.00392

**Published:** 2017-12-01

**Authors:** Jessica I. Fleck, Julia Kuti, Jeffrey Mercurio, Spencer Mullen, Katherine Austin, Olivia Pereira

**Affiliations:** ^1^School of Social and Behavioral Sciences, Stockton University, Galloway Township, NJ, United States; ^2^Department of Cell Biology and Molecular Biology, National Institute of Child Health and Human Development, Bethesda, MD, United States; ^3^School of Graduate Studies, Stockton University, Galloway Township, NJ, United States; ^4^Department of Biomedical Research, Nemours Hospital for Children, Wilmington, DE, United States

**Keywords:** cognitive reserve, resting-state EEG, aging, global coherence, cognitive function

## Abstract

Cognitive reserve (CR) is a protective mechanism that supports sustained cognitive function following damage to the physical brain associated with age, injury, or disease. The goal of the research was to identify relationships between age, CR, and brain connectivity. A sample of 90 cognitively normal adults, ages 45–64 years, had their resting-state brain activity recorded with electroencephalography (EEG) and completed a series of memory and executive function assessments. CR was estimated using years of education and verbal IQ scores. Participants were divided into younger and older age groups and low- and high-CR groups. We observed greater left- than right-hemisphere coherence in younger participants, and greater right- than left-hemisphere coherence in older participants. In addition, greater coherence was observed under eyes-closed than eyes-open recording conditions for both low-CR and high-CR participants, with a more substantial difference between recording conditions in individuals high in CR regardless of age. Finally, younger participants low in CR exhibited greater mean coherence than younger participants high in CR, whereas the opposite pattern was observed in older participants, with greater coherence in older participants high in CR. Together, these findings suggest the possibility of a shift in the relationship between CR and brain connectivity during aging.

## Introduction

Maintaining cognitive function is essential to achieve a high quality of life during the transition into older age. Researchers have demonstrated the important role of lifestyle factors, including diet and exercise, monitoring primary health concerns, and maintaining a stimulating environment, both cognitively and socially (see Ruthirakuhan et al., 2012) in sustained cognition. Life experiences, including education, occupational attainment, and dynamic social networks can alter cognitive function through cognitive reserve (CR; Stern, 2002).

Cognitive reserve is the ability of the individual to sustain cognition in spite of age- or disease-related changes to the physical brain (e.g., the accumulation of amyloid beta in Alzheimer’s disease; Stern, 2002, 2009; Rentz et al., 2010). CR can be acquired throughout the lifetime by participating in enriching cognitive and social activities (Stern, 2002), and is malleable into older age (Lenehan et al., 2016). CR differs from brain reserve, a form of reserve in which the physical properties of the brain (e.g., brain volume and the number of connections among neurons) influence cognitive outcomes (Tucker and Stern, 2011). In a recent review of animal studies that examined the effects of enriched housing on brain reserve, Gelfo et al. (2017) highlighted the ability of enriched environments to increase levels of neurogenesis and synaptogenesis in the brain, thereby enhancing the brain’s potential for plasticity. Both CR and brain reserve are thought to influence the resilience of the individual to age-related and clinical decline (see Stern, 2002; Medaglia et al., 2017).

Though the neural mechanisms that underlie CR are currently unknown, it has been proposed that CR supports sustained cognition through neural reserve (i.e., more efficient processing; Speer and Soldan, 2015), and neural compensation (i.e., the incorporation of alternate brain networks when primary networks are compromised; see Steffener and Stern, 2012, for a review). The positive impact of CR on cognition extends beyond its influence on individuals with Alzheimer’s disease (AD) and related dementias to those who have experienced traumatic brain injury, vascular disease, and neurodegenerative diseases (Tucker and Stern, 2011). CR has also been linked to positive clinical outcomes by reducing the risk of late-onset depression in older adults (Freret et al., 2015), enhancing the importance of research in this area.

Prior research has linked increased CR to improved cognitive function and altered task-related brain activity in cognitively normal older adults and individuals with AD and mild cognitive impairment (MCI). Healthy older adults with higher levels of education and occupational attainment, proxies used to estimate CR, demonstrate stronger performance on measures of memory, verbal fluency, and processing speed (e.g., Manly et al., 2005; Fritsch et al., 2007), as well as reduced brain activity during cognitive tasks (e.g., Bartrés-Faz et al., 2009; López et al., 2014). In contrast, high CR individuals with AD and MCI exhibit greater task-related brain activity than their low-CR counterparts (e.g., Scarmeas et al., 2004; Bosch et al., 2010; Steffener et al., 2011) and in some cases display activity in compensatory networks (e.g., Celone et al., 2006). High levels of CR have also been shown to delay the onset of cognitive symptoms associated with the physical pathology of AD (Steffener and Stern, 2012). These patterns suggests that CR provides a mechanism for neural efficiency during task-directed cognition in normal aging, along with an added resource for neural compensation during cognitive decline (see Barulli and Stern, 2013, for a review).

Beyond task-directed brain activity, researchers have begun to explore the relationship between activity in the brain at rest and CR (e.g., Bastin et al., 2012; Marques et al., 2016; Franzmeier et al., 2017c). Resting-state brain activity is recorded when the individual is in a relaxed and awake state, but not engaged in task-directed cognition. Exploration of the resting state is important as it provides information on overall brain organization independent of cognitive task demands (Stam, 2014). Across the spectrum of AD, from preclinical to dementia stages, higher CR is associated with lower FDG-PET metabolism, signifying greater levels of brain pathology in high-CR participants, when compared to individuals of similar diagnosis and cognitive ability who are low in CR (e.g., Ewers et al., 2013; Morbelli et al., 2013). Moreover, researcher have identified differences between high- and low-CR participants in resting-state network connectivity using functional MRI. For example, Franzmeier et al. (2017a) observed that high-CR patients with MCI possessed more brain voxels with high global brain connectivity, whereas low-CR participants displayed more brain voxels with low global brain connectivity. In addition, CR level has been shown to influence the patterns of activity among brain networks (Franzmeier et al., 2017b). Patients with MCI who were high in CR displayed the expected inverse pattern of activation between the dorsal attention network (DAN) and the default mode network (DMN; the DAN is highly active during task-directed cognition and the DMN during self-referential processing), whereas patients with low CR showed a reduction in this distinction.

In addition to the global brain connectivity differences noted above, researchers have indicated that connectivity within the left lateral prefrontal cortex (LPFC) may be uniquely important as an indicator of CR and predictor of cognitive function (e.g., Cole et al., 2012; Franzmeier et al., 2017c). Using resting-state functional MRI, Cole et al. (2012) identified the left LPFC, a subregion of the frontoparietal control network, as an important region for CR in healthy adults. Participants with the greatest global connectivity between the left LPFC cortex and other brain regions exhibited the highest levels of fluid intelligence. Moreover, Franzmeier et al. (2017c) reported that higher levels of CR were associated with greater connectivity between the left frontal region and the DAN, whereas lower levels of CR were associated with greater connectivity between the left frontal region and the DMN. These patterns were observed in cognitively normal older adults, as well as in older adults with MCI. Further, for both patients and controls, greater connectivity within the left frontal region during successful memory encoding was associated with higher levels of CR (Franzmeier et al., 2017d).

Considered together the above findings describe a decline in resting-state brain activity and cognitive function in conjunction with AD and MCI, but suggest that CR may influence the impact of physiological decline on cognition. If so, understanding how CR influences activity in the brain at rest, as well as the patterns of resting-state activity that are associated with high CR, are important steps toward understanding the neural underpinnings of CR. Preliminary research using functional MRI suggests that CR may change the impact of physical decline on cognition by influencing brain network connectivity (e.g., Franzmeier et al., 2017b). To our knowledge, no prior research has explored differences in resting-state connectivity in relation to CR, using EEG. However, Miraglia et al. (2017) have suggested that network analyses with EEG may play an important role in identifying and understanding individual differences in risk for clinical decline. Further, because EEG is less expensive and more easily tolerated than other measures of brain activity, it has the potential for widespread clinical application.

Numerous differences in brain activity distinguish older and younger participant groups, thus rendering age an important consideration in research exploring the influence of CR on resting-state activity. Healthy older adults demonstrate lower task-related activity than younger adults (e.g., Cabeza et al., 2004; Grady et al., 2006). In addition, research exploring age-related changes in resting-state activity has determined that connectivity within resting-state networks (e.g., DMN and attention networks) decreases during aging (see Ferreira and Busatto, 2013; Sala-Llonch et al., 2015). However, increases in local connectivity have been recorded within frontal regions (Davis et al., 2008; Turner and Spreng, 2012) and in some cases parietal regions (Toussaint et al., 2011). In younger adults, higher IQ scores have been associated with reduced resting-state network connectivity when compared to younger adults with lower IQ scores (e.g., Thatcher et al., 2005; Cheung et al., 2014), perhaps evidence of neural efficiency. Interestingly, a similar pattern was observed between CR and brain connectivity in a study of older adults, in which Bastin et al. (2012) observed lower activity in resting-state networks in participants who were high in CR, which may indicate that increased CR is associated with improved network efficiency throughout the course of healthy aging.

The association between brain connectivity and cognitive function (e.g., Sala-Llonch et al., 2015; Fleck et al., 2016; Vecchio et al., 2016), as well as the influence of CR on task-related brain activity and cognition (e.g., Scarmeas et al., 2004; Bosch et al., 2010), suggests that CR may influence functional connectivity in the brain at rest. In the present research, we used electroencephalography (EEG) to explore differences stemming from age and CR in resting-state coherence in adults ages 45–64 years. EEG coherence is a measure of the synchronous activity among brain regions and is indicative of underlying network connectivity (Thatcher, 2012). We targeted participants between 45 and 64 years of age because of the importance that has been placed on the preclinical period as a critical window for the early detection of cognitive change during aging (Sperling et al., 2011). To begin, we examined the relationships between age and global brain connectivity, measured using mean EEG coherence (see Babiloni et al., 2009), calculated separately for high-CR and low-CR groups. Based on these results, we then divided the sample into younger and older age groups and explored differences in global brain connectivity among participants in the four different age/CR conditions. Research has suggested that brain activity during eyes-open versus eyes-closed recording conditions captures different brain states (see Miraglia et al., 2016, for a review), with eyes-closed recordings capturing internally directed thought and eyes-open recordings capturing attention and stimulus processing (Marx et al., 2004). Thus, it was possible that CR level would be associated with different outcomes based on recording condition. Therefore, we recorded resting-state EEG under eyes-open and eyes-closed recording conditions. Although our research was largely exploratory, for younger sample members we predicted that individuals high in CR would show lower global brain coherence than individuals low in CR, similar to the inverse relationship between IQ and brain connectivity reported for younger adults in prior research (Thatcher et al., 2005; Cheung et al., 2014). However, for older sample members we predicted that participants high in CR would show greater global brain coherence than participants low in CR, reflecting the positive impact of CR in limiting the decline in resting-state network connectivity that accompanies aging (see Ferreira and Busatto, 2013, for a review).

## Materials and Methods

### Participants

Data were collected from 93 participants from southern New Jersey. Participants were volunteers recruited via newspaper advertisement (they were not paid for their involvement), were right handed (as verified by scores on the Edinburgh Handedness Inventory; Oldfield, 1971), had normal or corrected hearing and vision, and no prior history of dementia. To increase the likelihood that we were testing adults experiencing normal aging, we excluded participants who self-reported any history of traumatic brain injury, stroke, or neurological disorder; two or more concussions; a history of drug or alcohol abuse; or the current use of medications for the treatment of anxiety or depression. Further, we excluded participants with scores less than 26 on the Mini Mental State Examination – 2nd Edition (MMSE-2; Folstein et al., 2010). Scores less than 26 on the MMSE are generally considered indicative of clinical decline (e.g., Vertesi et al., 2001). After applying the above criteria, data from 90 participants (58 female) were retained for analysis. Mean age of the sample was 58.51 years (*SD* = 4.37) and 87.88% of the sample was Caucasian.

### Materials

#### Electroencephalography

Electroencephalography data were recorded using a 129-channel HydroCel Geodesic Sensor Net, with Cz reference (Electrical Geodesics, Inc.). Sensor impedance levels were below 50 KΩ, appropriate for use with the Net Amps 300 high-impedance amplifier. Data were sampled at 250 Hz, and filtered using an analog.1 – 100 Hz bandpass filter. Three minutes of eyes-open data followed by 3 min of eyes-closed data were recorded from each participant using Net Station 4.2 software. Data from the 19 channels in the 10–20 electrode system of placement were exported from Net Station for artifact removal and data reduction using NeuroGuide 2.6.5 (Applied Neuroscience, Inc.; Thatcher, 2015). Data were re-referenced to linked mastoids and each participant’s EEG record was visually inspected for artifact, with the first 90 s of clean EEG data in the eyes closed and eyes open blocks selected for additional processing.

All coherence calculations were performed in NeuroGuide for the following frequency bands: delta (1.0 – 4.0 Hz), theta (4.0 – 8.0 Hz), low alpha (8.0 – 10.0 Hz), high alpha (10.0 – 12.0 Hz), beta (12.5 – 25.0 Hz) and gamma (30.0 – 50.0 Hz) (see Thatcher, 2012). As described in Thatcher (2012), coherence was calculated as the spectral cross correlation between electrodes, normalized by the electrodes’ power spectra.

#### Neuropsychological Measures

The neuropsychological battery administered in the present research contained the MMSE-2 (Folstein et al., 2010), The Clock Drawing Test (Strauss et al., 2006), a measure of verbal intelligence, and several measures of memory and executive function. The National Adult Reading Test – Revised (NART-R; Blair and Spreen, 1989) was used in the present research to estimate verbal IQ. The NART-R contains 61 words with non-phonetic spellings. Participants are asked to read the words aloud. The number of incorrectly pronounced words is used to estimate the participant’s verbal IQ.

To assess memory function, the Digit Span subtest of the Wechsler Adult Intelligence Scale-Fourth Edition (WAIS – IV; Wechsler, 2008) and the California Verbal Learning Test – Second Edition (CVLT-II; Delis et al., 2000) were administered. For the Digit Span, a measure of working memory, individuals were read a series of numbers and were asked to repeat those numbers in the same order (forward), in reverse order (backward) or in ascending order (sequencing), for the respective sections of the assessment. In contrast, the CVLT-II (Delis et al., 2000) was administered as an assessment of long-term memory. Individuals were asked to learn a list of 16 words from four categories in five trials, with each trial including list presentation and list recall, and to subsequently recall those words after a brief distractor task (short-term recall) and again after a 20-min delay (long-term recall). For the current research, the number of items recalled during the encoding phase for trials 1 – 5 were summed to generate a CVLT-II Trials I-V score. In addition, the total number of list items recalled after the 20-min delay (CVLT-II Delayed Recall) was included as a measure of long-term memory.

To determine participants’ abilities in flexible thinking, strategy use, and related processes, participants completed the Delis-Kaplan Executive Function System (D-KEFS) Verbal Fluency Test (Delis et al., 2001), as well as The Trail Making Test (Reitan and Wolfson, 1993). For Verbal Fluency, participants were asked to generate as many words as possible that began with a specific letter (i.e., Letter Fluency), and were asked to generate as many category members as possible (i.e., Category Fluency), with 60 s per trial. The total number of unique, correct responses were summed to generate total scores for Letter Fluency and Category Fluency. In the Trail Making Test (Reitan and Wolfson, 1993) individuals are asked to connect, in numerical order, encircled numbers that are randomly presented on a page (i.e., Trails A) and then, to connect encircled numbers and letters in alternating order (e.g., 1 to A, A to 2, 2 to B; Trails B). Trails A and Trails B completion times were used as performance indicators.

### Procedure

The research procedure for the project was approved by Stockton University’s Institutional Review Board. Participants completed two research sessions each lasting 1 – 1.5 h in duration, scheduled 1–2 weeks apart. All participants provided written informed consent and then completed the EEG recording and self-report measures as part of Session 1. During the EEG recording, participants had their eyes-open and eyes-closed, resting-state EEG activity recorded for 3 min each. Prior to the recording, participants were asked to sit in a relaxed position and to keep their minds free from other thoughts. Participants were visually monitored for adherence to the instructions, as well as drowsiness during the recording session.

The battery of neuropsychological measures was administered during Session 2. All participants completed the neuropsychological measures in the same order: (a) MMSE-II, (b) Digit Span, (c) CVLT-II, (d) Trails A and B, (e) NART-R, (f) The Clock Drawing Test, (g) CVLT-II – 20-min delayed recall and recognition, and (h) Verbal Fluency. After completing the assessments, participants were debriefed, thanked for their participation, and the session concluded. We note that participants who scored 2.0 or more standard deviations below their age-appropriate mean on any one assessment, or 1.5 standard deviations below their age-appropriate mean on two or more assessments, were sent a letter recommending a follow-up assessment in the community.

## Results

Cognitive reserve was calculated for each participant by creating a composite variable using estimated verbal IQ score (NART-R) and years of education. Participants’ scores on IQ and education were Z-transformed and averaged to form the composite score. We observed no violations of normality nor the presence of outliers when we examined the composite scores for normality. Participants were divided into CR groups (low-CR; *n* = 43, high-CR; *n* = 47) using a median split. The unequal split between conditions arose because three participants had z scores of 0.08 and were all placed in the high-CR group. Descriptive statistics for key demographic and neuropsychological variables are presented separately by CR group in **Table 1**. Prior to analysis, we screened all neuropsychological variables for violations of normality and linearity, as well as for the presence of univariate and multivariate outliers. One univariate outlier on the Trails B assessment (Delis et al., 2001) was addressed using pairwise deletion. No multivariate outliers were identified using Mahalanobis distance (*p* < 0.001). No other violations were detected.

**Table 1:** Descriptive statistics (means and standard deviations) for neuropsychological measures by CR group.

	Low CR	High CR
*n*	43	47
Age	58.72 (4.11)	58.32 (4.62)
Education	13.56 (1.91)	17.38 (1.62)
Verbal IQ	109.62 (5.11)	118.24 (4.82)
MMSE	28.21 (1.54)	29.00 (1.10)
Digit span total	26.26 (5.15)	29.36 (4.28)
CVLT total recall	46.72 (7.88)	50.83 (9.90)
CVLT delayed recall	10.16 (2.95)	11.62 (3.00)
Trails A	26.82 (7.34)	26.60 (7.91)
Trails B	62.96 (29.82)	59.68 (17.51)
Letter fluency	40.60 (10.84)	48.91 (9.61)
Category fluency	42.23 (7.94)	45.60 (6.17)

We separately calculated mean intrahemispheric coherence for electrode pairs in the left hemisphere and the right hemisphere, allowing us to focus on differences in coherence between hemispheres for high-CR and low-CR groups. Mean coherence was separately calculated for each frequency band (delta, theta, low alpha, high alpha, beta, and gamma) under eye-closed and eyes-open recording conditions.

To explore potential differences in cognitive performance between CR groups (low-CR, high-CR), a between-subjects multivariate analysis of variance was performed using the eight neuropsychological assessment scores listed in **Table 2** as dependent variables. Using Wilks’ Lambda, the combined dependent variables were significantly affected by CR, *F*(8, 77) = 3.635, *p* = 0.001, $\eta_{p}^{2}$ = 0.274. To explore differences between CR groups for individual dependent variables, univariate ANOVAs were conducted separately for each neuropsychological assessment (see **Table 2**). Using a corrected alpha level of 0.00625 (0.05/8 neuropsychological tests), a significant difference in performance was observed between CR conditions for MMSE, Digit Span Total, and Fluency. In all instances, high-CR participants exhibited stronger cognitive performance than low-CR participants. There was no significant difference between CR conditions for age, *F*(1,88) = 0.188, *p* = 0.665, $\eta_{p}^{2}$ = 0.002.

**Table 2:** Univariate ANOVA results comparing low-CR and high-CR groups on neuropsychological measures.

Measure	*F*	*P*	*R*^2^
MMSE	8.749	0.004^∗^	0.094
Digit span total	9.098	0.003^∗^	0.098
CVLT total recall trials I-V	5.025	0.028	0.056
CVLT 20-min delayed recall	6.091	0.016	0.068
Trails A	0.077	0.782	0.001
Trails B	0.395	0.531	0.005
Letter Fluency	13.731	<0.001^∗^	0.140
Category fluency	5.139	0.026	0.058

^∗^*Significant at the corrected alpha level of 0.00625*.

**FIGURE 1:**
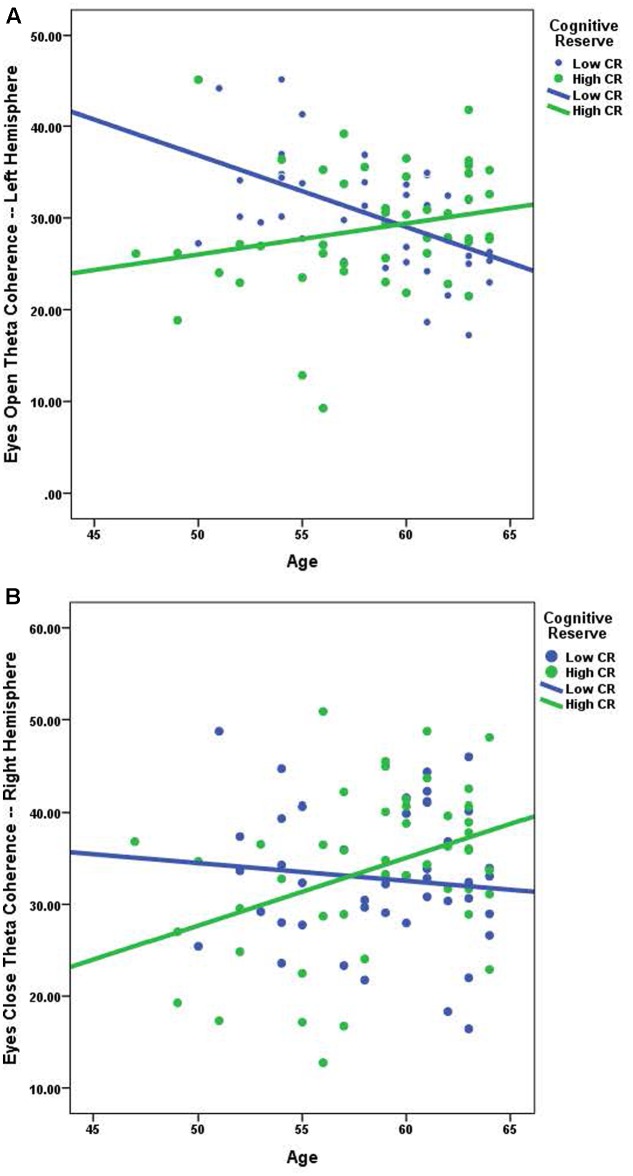
**(A)** The relationship between age and eyes open theta coherence — left hemisphere; low CR: *r* = -0.526, high CR: *r* = 0.231. **(B)** The relationship between age and eyes closed theta coherence – right hemisphere; low CR: *r* = -0.107, high CR: *r* = 0.385.

Using Pearson correlations, we examined the relationship between age and coherence separately for high-CR and low-CR groups under eyes-closed and eyes-open recording conditions (see **Table 3**). Correlations were calculated for each frequency band and all analyses were conducted as two-tailed tests, using a corrected alpha of 0.0083 (0.05/6 frequency bands). Relationships marked as significant at the 0.01 level in **Table 3** also met the 0.0083 corrected alpha level for significance. For low-CR participants, we observed significant inverse relationships between age and brain coherence over the left hemisphere, with most significant correlations present under eyes-open recording conditions. In contrast, for high-CR participants we detected significant positive relationships between age and coherence over the right hemisphere; these correlations were only statistically significant for the theta and high alpha frequency bands under eyes-closed recording conditions. As reflected in **Table 3**, most correlation coefficients between age and brain coherence were negative for low-CR participants, whereas most correlation coefficients between age and brain coherence were positive for high-CR participants. The relationships between age and coherence are plotted together for the two CR groups in **Figures 1A,B**.

**Table 3:** Correlations between age and resting-state EEG coherence.

	Low CR				High CR			
	Eyes closed		Eyes open		Eyes closed		Eyes open	
	LH	RH	LH	RH	LH	RH	LH	RH
Delta	-0.245	-0.171	-0.388^∗^	-0.192	0.082	0.341^∗^	0.052	0.172
Theta	-0.332^∗^	-0.107	**-0.526^∗∗∗^**	-0.167	0.234	**0.385^∗∗^**	0.231	0.289^∗^
Low alpha	-0.184	0.184	**-0.413^∗∗^**	-0.038	0.104	0.194	0.130	0.288
High alpha	-0.254	-0.200	-0.319^∗^	-0.089	0.103	**0.403^∗∗^**	0.033	0.253
Beta	-0.330^∗^	-0.141	**-0.429^∗∗^**	-0.194	0.114	0.302^∗^	0.025	0.183
Gamma	**-0.402^∗∗^**	-0.255	**-0.493^∗∗∗^**	-0.313	0.212	0.234	0.069	0.150

^∗^*p < 0.05, ^∗∗^*p* < 0.01, ^∗∗∗^*p* < 0.001. Correlations in bold are significant following alpha correction*.

In order to more fully examine the complex relationships between age, CR, and brain connectivity presented in **Table 3** and **Figures 1A,B**, we divided participants into high and low age groups using a mean split. Participants below the mean age of 58.51 years were placed in the younger group. We then conducted six mixed model ANOVAs, one for each frequency band, exploring differences between Age and CR groups in global brain connectivity. Age and CR were between-subjects variables in the design and hemisphere (left, right), and recording condition (eyes-closed, eyes-open) were within-subjects variables. Descriptive statistics for coherence are reported by condition for the left and right hemispheres, under eyes-closed and eyes-open recording conditions in **Table 4**. Because of our interest in understanding the impact of age and CR on coherence, we focused on significant interactions that included age, CR, or both age and CR as variables. Significant main-effects or interactions involving only within-subjects variables are not reported. For all mixed model ANOVAs we used a corrected alpha of 0.0083 (0.05/6 frequency bands); significant interaction results are summarized in **Table 5**.

**Table 4:** Coherence descriptive statistics, means (standard deviations).

	Younger		Older	
	Low CR	High CR	Low CR	High CR
**Delta**				
Closed				
Left	28.34 (5.88)	28.76 (10.80)	25.32 (5.85)	28.81 (6.06)
Right	28.08 (7.66)	24.25 (8.07)	26.71 (7.94)	31.67 (6.91)
Open				
Left	28.89 (6.14)	26.86 (10.62)	24.50 (5.28)	26.75 (6.12)
Right	27.50 (6.79)	24.10 (9.02)	26.07 (6.66)	29.26 (7.60)
**Theta**				
Closed				
Left	33.94 (5.50)	31.48 (7.95)	29.47 (7.03)	33.58 (7.29)
Right	32.09 (7.46)	29.32 (9.82)	32.99 (7.52)	37.60 (6.00)
Open				
Left	33.68 (5.76)	27.57 (8.42)	27.36 (5.06)	29.98 (4.97)
Right	32.50 (9.25)	27.56 (9.30)	31.88 (6.93)	34.23 (6.41)
**Low alpha**				
Closed				
Left	40.94 (7.23)	40.80 (7.66)	36.22 (8.01)	40.87 (8.79)
Right	33.01 (7.66)	37.78 (9.51)	38.22 (8.66)	42.28 (8.19)
Open				
Left	37.55 (7.97)	32.01 (7.87)	30.69 (5.33)	33.24 (6.04)
Right	32.19 (7.42)	30.32 (8.75)	34.65 (8.32)	36.24 (6.37)
**High alpha**				
Closed				
Left	37.11 (4.00)	37.77 (7.94)	33.02 (7.80)	38.79 (7.00)
Right	34.21 (7.58)	33.73 (8.24)	33.97 (6.04)	39.86 (5.64)
Open				
Left	35.02 (5.76)	31.08 (8.82)	29.85 (6.12)	32.01 (5.48)
Right	31.88 (7.69)	28.93 (7.10)	32.64 (7.12)	35.05 (6.47)
**Beta**				
Closed				
Left	30.59 (5.79)	29.91 (7.11)	27.04 (5.17)	32.57 (6.77)
Right	29.67 (9.03)	27.21 (6.00)	29.60 (6.41)	33.72 (5.92)
Open				
Left	29.46 (8.37)	25.06 (8.01)	24.10 (5.32)	26.11 (6.07)
Right	29.17 (10.88)	22.64 (6.42)	26.98 (7.34)	28.41 (8.92)
**Gamma**				
Closed				
Left	29.37 (11.98)	25.22 (8.91)	22.26 (5.38)	31.52 (13.95)
Right	30.28 (16.78)	22.05 (5.74)	25.20 (8.99)	30.84 (11.87)
Open				
Left	28.56 (11.37)	22.10 (9.38)	20.49 (6.10)	24.19 (10.63)
Right	30.41 (15.58)	17.32 (4.55)	23.37 (10.87)	23.03 (12.30)

**Table 5:** Significant ANOVA interactions showing differences in EEG coherence between age and CR conditions.

	*F*	*p*	*R*^2^
**Delta**			
Age ^∗^ Hemisphere	8.771	0.004	0.093
**Theta**			
CR ^∗^ Recording condition	10.514	0.002	0.109
Age ^∗^ Hemisphere	10.486	0.002	0.109
Age ^∗^ CR	9.336	0.003	0.098
**Low alpha**			
CR ^∗^ Recording condition	10.313	0.002	0.107
Age ^∗^ Hemisphere	17.341	<0.001	0.168
**High alpha**			
CR ^∗^ Recording condition	7.626	0.007	0.081
Age ^∗^ Hemisphere	11.63	0.001	0.119
Age ^∗^ CR	7.608	0.007	0.081
**Beta**			
CR ^∗^ Recording condition	24.685	<0.001	0.223
Age ^∗^ CR	7.295	0.008	0.078
**Gamma**			
CR ^∗^ Recording condition	18.229	<0.001	0.175
Age ^∗^ CR	10.237	0.002	0.106

**FIGURE 2:**
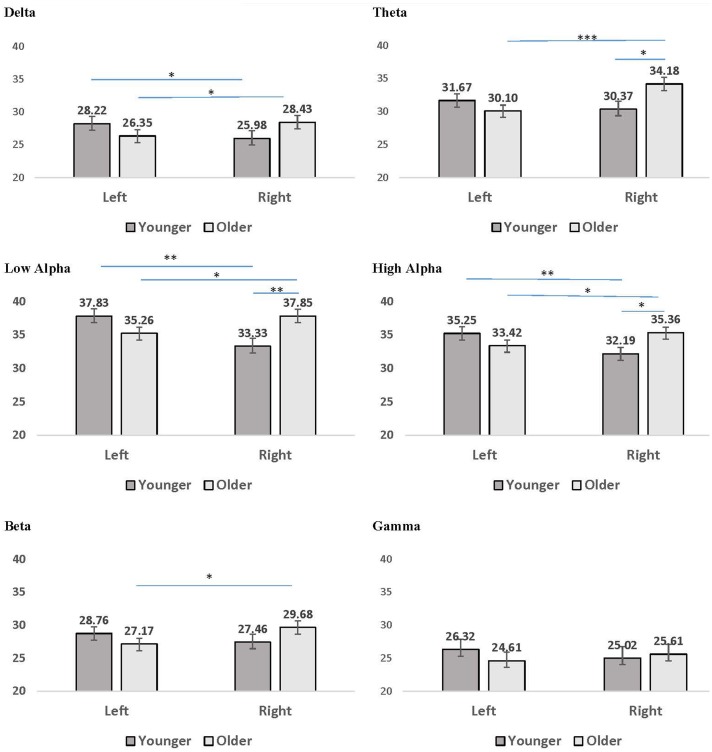
Mean coherence within the left hemisphere and right hemisphere for younger (dark gray bars) and older (light gray bars) participant groups. Error bars reflect the standard error for each condition. Lines connect conditions that differ significantly from each other, with the endpoints of each line over the middle of the bars for conditions that differ significantly from each other during pairwise comparisons. ^∗^*p <* 0.05, ^∗∗^*p* < 0.01, ^∗∗∗^*p* < 0.001.

Several interaction patterns emerged as a result of our analyses. First, significant age by hemisphere interactions were observed for all frequency bands, from delta through high alpha, but failed to reach significance for beta or gamma frequencies. Least significant difference (LSD) *post hoc* analyses were conducted within each frequency band to clarify the nature of the interactions (see **Figure 2**). For younger participants left-hemisphere coherence was greater than right-hemisphere coherence for delta, low alpha, and high alpha frequencies. In contrast, for older participants right-hemisphere coherence was greater than left-hemisphere coherence for all frequency bands, for delta through high alpha. In addition, comparisons between age groups conducted separately by hemisphere revealed greater right-hemisphere coherence for older participants than for younger participants in theta, low alpha, and high alpha frequency bands. No significant differences were observed between age groups for left-hemisphere coherence. The three-way interaction for age, CR, and hemisphere failed to reach significance for any of the frequency bands, suggesting no significance difference in the above patterns between CR groups.

**FIGURE 3:**
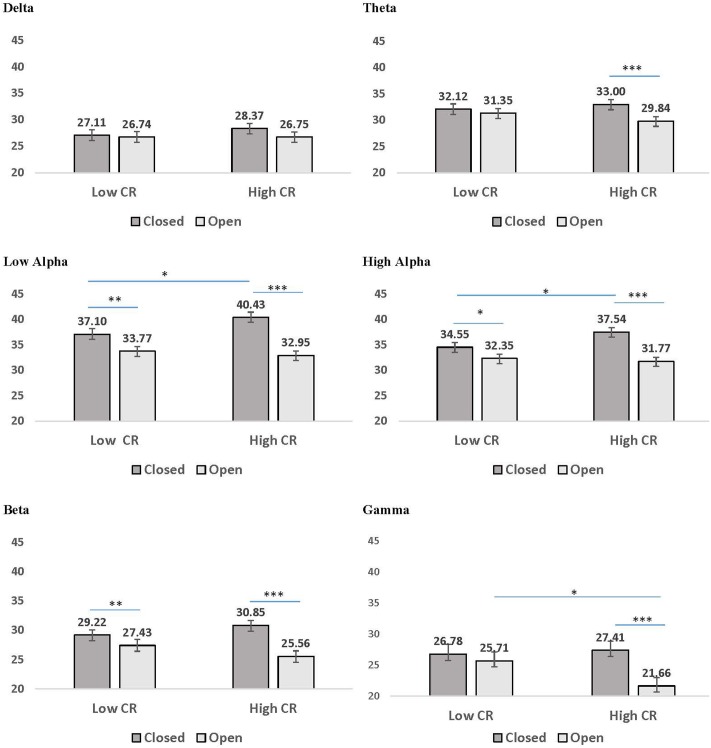
Mean coherence in low CR and high CR participants for eyes-closed (dark gray bars) and eyes-open (light gray bars) recording conditions. Error bars reflect the standard error for each condition. Lines connect conditions that differ significantly from each other, with the endpoints of each line over the middle of the bars for conditions that differ significantly from each other during pairwise comparisons. *^∗^p <* 0.05, ^∗∗^*p* < 0.01, ^∗∗∗^*p* < 0.001.

A second pattern within the interaction results included significant CR by recording condition interactions for all frequency bands except delta. LSD *post hoc* analyses were conducted for each frequency band and the results are presented in **Figure 3**. In low-CR participants, greater coherence was exhibited for eyes-closed than eyes-open recording conditions, for low alpha, high alpha, and beta frequencies. The same pattern was exhibited for high-CR participants; however, the increase in coherence from eyes-open to eyes-closed recordings for high-CR participants were more substantial than that exhibited by low-CR participants, and extended from theta through gamma frequencies. Comparisons conducted separately by recording condition identified greater eyes-closed coherence for high-CR than low-CR participants for low alpha and high alpha frequency bands, and greater eyes-open coherence for low-CR than high-CR participants in the gamma frequency band. The three-way interaction for age, CR, and recording condition failed to reach significance for any of the frequency bands, suggesting no significant difference in the patterns between age groups.

**FIGURE 4:**
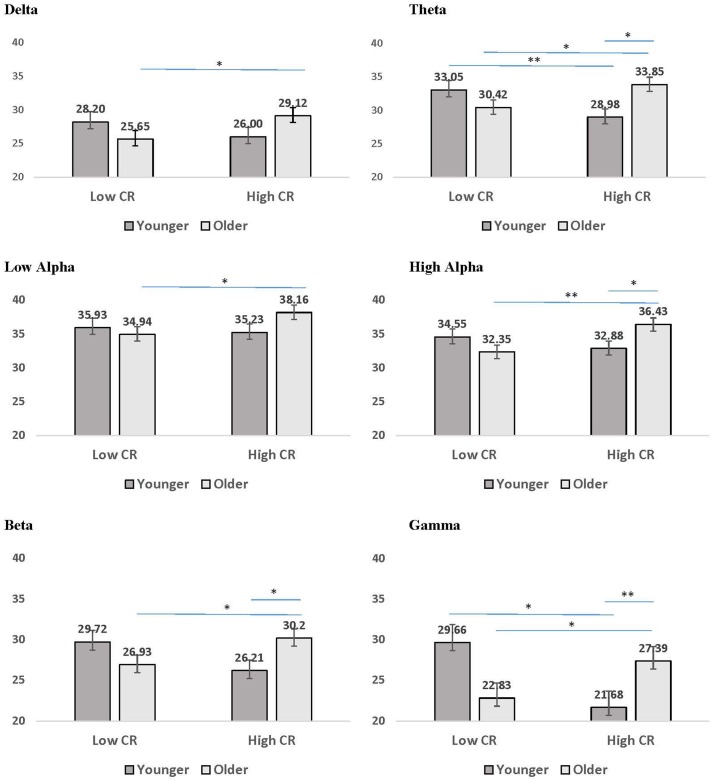
Mean coherence for younger (dark gray bars) and older (light gray bars) participants in low CR versus high CR conditions. Error bars reflect the standard error for each condition. Lines connect conditions that differ significantly from each other, with the endpoints of each line over the middle of the bars for conditions that differ significantly from each other during pairwise comparisons. ^∗^*p <* 0.05, *^∗∗^p <* 0.01, ^∗∗∗^*p* < 0.001.

The third pattern that emerged in the interaction data included significant age by CR interactions for mean coherence, present in all frequency bands except delta and low alpha. The results of LSD *post hoc* analyses conducted for each frequency band are presented in **Figure 4**. For younger participants, mean coherence was greater in low-CR than in high-CR participants for theta and gamma frequency bands. In contrast, for older participants mean coherence was greater in high-CR than in low-CR participants for all frequency bands. Further comparisons of age differences within CR groups identified greater mean coherence in older than in younger participants who were high in CR for theta, high alpha, beta, and gamma frequency bands. No significant differences were observed between older and younger participant groups who were low in CR.

We also explored our dataset for possible gender differences in age, CR, and EEG coherence. In doing so, we failed to find significant differences between men and women in age or CR [age: *F*(1,88) = 0.342, *p* = 0.560, $\eta_{p}^{2}$ = 0.004; CR: *F*(1,88) = 0.592, *p* = 0.444, $\eta_{p}^{2}$ = 0.007]. To test for possible gender differences in global brain coherence, we conducted six 2 × 2 × 2 mixed-model ANOVAs with gender as a between-subjects variable and hemisphere and recording condition as within-subjects variables. Gender differences were observed for the high alpha frequency band, with greater coherence in women than men [*F*(1,88) = 7.480, *p* = 0.008, $\eta_{p}^{2}$ = 0.078; women: *M* = 35.16, *SEM* = 0.65; men: *M* = 32.21, *SEM* = 0.87] but all other frequencies failed to achieve statistical significance (*p* > 0.05). To determine if the significant gender difference in the high alpha frequency band affected the interactions observed for age and CR reported above, we conducted a mixed model ANOVA with age, CR, and gender as between-subjects variables and hemisphere and recording condition as within-subjects variables. However, there were no significant interactions involving gender with any other variables in the design (*p* > 0.05).

## Discussion

The goal of the present research was to identify differences in resting-state EEG coherence associated with CR level and age in adults, ages 45–64 years. Global coherence differences emerged between age groups for left- versus right-hemisphere connectivity and between CR groups for eyes-closed versus eyes-open recording conditions. In addition, age-CR interactions showed differences in scalp-wide coherence, revealing important effects of age and CR on resting-state connectivity. In this interaction, younger participants low in CR exhibited greater EEG coherence than younger participants high in CR, whereas older participants high in CR demonstrated greater EEG coherence than older participants low in CR. These findings are discussed in turn below.

Hemispheric differences in coherence were evident between older and younger participant groups, with greater left- than right-hemisphere coherence in younger participants and greater right- than left-hemisphere coherence in older participants. The source of the age-related shift in the present research was driven by the right hemisphere, with greater coherence in the right-hemisphere for older than younger participants for theta and alpha frequency bands, but the absence of significant differences between age groups in left-hemisphere coherence. Additionally, we observed an increase in right-hemisphere coherence with age in high-CR participants. An increase in bilateral processing, along with a decrease in region-specific processing in various cognitive domains has been identified in conjunction with aging (see Grady, 2012, for a review). Further, Antonenko and Flöel (2014) suggest that a reduction in lateralized processing in aging is a contributor to decline in cognitive function, with the successful execution of many lateralized processes, such as language, by younger adults associated with increased connectivity within the left hemisphere (Antonenko et al., 2013).

While an increase in right-hemisphere connectivity with aging may reflect a loss of functional and neural specificity, as noted above, it has also been proposed that an increase in right-hemisphere connectivity is a positive outcome associated with high CR. Robertson (2013, 2014) has suggested that the right LPFC and the right inferior parietal lobe may be uniquely important in CR. According to Robertson’s theory, CR leads to an increase in the neurotransmitter noradrenaline (NA), which is an important neurotransmitter in arousal, sustained attention, working memory, and related domain-general processes. Further, positive correlations have been observed between NA neuronal density in the locus ceruleus and cognitive function during the final 5 years of life (Wilson et al., 2013). Empirical research directly testing the role of the right hemisphere in CR revealed faster processing speeds during a whole report task in response to stimuli presented in the left visual field for participants high in CR (Brosnan et al., 2017). Furthermore, when the researchers increased activation in the right prefrontal cortex using transcranial direct current stimulation processing speed improved in low CR participants, specifically for left visual field stimuli (Brosnan et al., 2017). Although we failed to observe significant three-way interactions involving age, CR, and hemisphere, the coherence data presented in **Table 3** may offer support for Robertson’s theory. As detailed in **Table 3**, we observed significant correlations between age and right-hemisphere coherence for high-CR participants in theta and high alpha frequency bands. Therefore, it is possible that high CR increases right-hemisphere connectivity during aging and enables better cognitive performance through the influence of CR on NA circuitry.

Beyond the hemispheric difference in coherence between age groups noted above, variations were observed in EEG coherence under eyes-closed versus eyes-open recording conditions between low-CR and high-CR groups. We observed greater connectivity under eyes-closed than eyes-open recording conditions for both low-CR and high-CR participants, with a more substantial difference in coherence between recording conditions for high-CR participants. These findings coincide with prior research by Knyazev et al. (2015) who reported decreased connectivity during eyes-open versus eyes-closed recording conditions for both younger and older participant groups. Prior research reporting greater alpha power under eyes-closed than eyes-open recording conditions observed a relationship between the degree of power change between recording conditions and measures of arousal, such as skin conductance level (Barry et al., 2007). We observed a more substantial decrease in alpha coherence from eyes-closed to eyes-open conditions in high-CR than in low-CR participants, which may reflect greater arousal in response to visual stimulation in conjunction with high CR. Therefore, although speculative, the differences between recording conditions in the present research may reflect the influence of CR on arousal level.

The most significant finding in our research is the interaction effect for age and CR on brain coherence. Theories of CR suggest that high levels of CR can benefit the individual through increased neural efficiency, as well as neural compensation (Stern, 2002, 2009). As an example, Speer and Soldan (2015) demonstrated that participants with high levels of CR demonstrated enhanced neural efficiency during a working memory task. In their research, younger adults and older adults completed a memory task, in which 1–7 letters were presented simultaneously, and recognition for the letters was tested after a brief delay. Participants who were high in CR showed a reduction in the electrical changes in the brain that typically occur during longer, more difficult trials in response to stimuli presented during the recognition phase (i.e., a reduction in amplitude decrease and less of an increase in latency of the Pb3 component). As noted by the researchers, higher levels of CR mitigated the neural changes associated with more difficult trials, demonstrating that high CR results in an increase in neural efficiency. Thus, Speer and Soldan’s findings offer support for the influence of CR on brain activity during task-directed cognition.

In the present research, differences in coherence between high-CR and low-CR groups varied for younger and older participant groups, with greater coherence in younger sample members who were low in CR and greater coherence in older sample members who were high in CR. Although resting-state EEG does not provide a direct measure of neural compensation and is a task-independent measure (i.e., a reduction in activity in task-typical networks coupled with an increase in activity in task-atypical networks is not discernable), there was evidence in the current research that higher levels of CR are associated with greater overall brain coherence in older participants. This increase was particularly evident in the right hemisphere in which high-CR older adults exhibited greater coherence than both low-CR older adults, and high-CR younger adults for all frequencies except delta and low alpha. Therefore, with aging, there is a shift in hemispheric dominance in coherence, which we suggest may reflect neural compensation in high-CR older adults in the present research.

Our correlation findings reporting the relationships between age and coherence within high-CR and low-CR groups may offer additional insight into how CR mitigates the changes in brain connectivity that occur in aging. Researchers have reported age-related reductions in resting-state network connectivity across brain networks, to include the DMN and FPAN (see Scheinost et al., 2015, for a review). We observed a significant decrease in resting-state coherence with aging in low-CR members of our sample under eyes-open recording conditions. This pattern coincides with prior research and may reflect a decrease in brain connectivity during aging, with the reduction in connectivity then influencing successful processing during externally directed cognition. In contrast, for high-CR participants, aging was associated with an increase in eye-closed connectivity. Because the eyes-closed resting state is thought to capture connectivity associated with internally directed cognition (see Miraglia et al., 2016), the age-related increase in eyes-closed connectivity for high-CR participants may reflect the influence of CR on DMN activity. Decreases in DMN have been reported during aging, with altered DMN connectivity reported in AD and MCI patient groups (e.g., Damoiseaux, 2012). Thus, the increase in eyes-closed connectivity with aging in high-CR participants may reflect the protective benefits of CR on this brain network.

We recognize several limitations with the present research. First, the majority of participants in the present research had attained a minimum education of a high school degree or its equivalent. This significantly truncated the range of possible CR scores in our sample and may limit our ability to estimate the impact of CR on brain coherence in the broader population. In addition, we estimated participants’ CR levels using a composite variable of verbal IQ and years of education. CR reviews have called for the comprehensive estimate of CR using proxies such as leisure activities and social network size (Jones et al., 2011), which are likely important contributors to the overall estimate of CR. The absence of additional CR proxies in the present research may have reduced the accuracy of our CR estimates. Further, we note that our sample was not comprised, in equal parts, of individuals representing all ages within our target age range of 45–64 years, with more sample members in the upper half than the lower half of this range. As a result, the age range in our younger participant group (45–58.10 years) was almost double of that in our older participant group (58.20–64 years). We feel it will be important for future research to increase the number of younger sample members to allow a more comprehensive assessment of the age variable so that changes in brain connectivity during middle age can be fully ascertained.

We also acknowledge several limitations to our resting-state EEG coherence data. First, our explorations of coherence failed to specifically assess changes in coherence between the hemispheres. In addition, connectivity in the brain at rest may offer an incomplete measure of the influence of CR on brain network connectivity. Individuals with higher levels of CR are believed to show the best performance on cognitive tasks, in spite of physical brain decline. Thus, it will be important for future research to compare the influence of CR on resting-state and task-directed brain activity measured from the same sample in order to clarify and how CR affects brain connectivity during each of these states.

Although the present research is the only research to our knowledge that has explored the effects of CR on resting-state EEG connectivity, we believe continued research in this area is invaluable. Certainly, in the absence of a cure for AD and related forms of dementia, lifestyle factors that mitigate cognitive changes associated with physiological decline in the brain are vital to enable cognitive health and independence during aging. Further, recent theories have suggested that CR may influence not only cognitive function in the face of physiological decline, but may alter the development of the disease pathology itself (see Arenaza-Urquijo et al., 2015). Therefore, understanding the neural underpinnings of CR, and how individual CR proxies are related to neural differences, may allow researchers and clinicians to subsequently utilize CR as a mechanism to alter brain connectivity and cognitive function.

## Ethics Statement

This study was carried out in accordance with the recommendations of the Stockton University Institutional Review Board with written informed consent from all subjects. All subjects gave written informed consent in accordance with the Declaration of Helsinki. The protocol was approved by the Stockton University Institutional Review Board.

## Author Contributions

JF developed the research design. JK, JM, SM, KA, and OP collected the associated data. JF, JK, and JM contributed to the analysis of the data, and all authors shared in drafting the manuscript.

## Conflict of Interest Statement

The authors declare that the research was conducted in the absence of any commercial or financial relationships that could be construed as a potential conflict of interest.
